# Evaluation of surgical outcomes in elderly patients with rib fractures: A single-centre propensity score matching study

**DOI:** 10.3389/fsurg.2023.1174365

**Published:** 2023-04-18

**Authors:** Dong Zhang, Chenbo Jiao, Siqi Xi, Langran Wang, Run Li, Qiang Zhang

**Affiliations:** ^1^ Department of Thoracic Surgery, Beijing Jishuitan Hospital, Beijing, China; ^2^ Health Science Center, Peking University, Beijing, China

**Keywords:** rib fracture, internal fixation, conservative treatment, trauma, pain, fracture union

## Abstract

**Background:**

Rib fractures are the most common injuries in chest trauma. Compared with younger patients, elderly patients with rib fracture have a higher incidence of complications and mortality. A retrospective study was conducted to investigate the effect of internal fixation compared with conservative treatment on the outcome of rib fracture in elderly patients.

**Material and methods:**

We used a 1:1 propensity score matching method to perform a retrospective analysis of 703 elderly patients with rib fractures treated in the Thoracic Surgery Department of Beijing Jishuitan Hospital between 2013 and 2020. After matching, the length of hospital stay, death, symptom relief and rib fracture healing were compared between the surgery and the control group.

**Results:**

The study included 121 patients receiving SSRF in the surgery group and 121 patients receiving conservative treatment in the control group. The length of hospital stay in the surgery group was significantly longer than that in the conservative group (11.39d vs. 9.48d, *p* = 0.000). After 9 months of follow-up, the fracture healing rate in the surgery group was significantly higher than that in the control group (96.67% vs. 88.89%, *p* = 0.020). The fracture healing time (*p* = 0.000), improvement in pain score (*p* = 0.000) and duration of pain medication use (*p* = 0.000) were also significantly better in the surgery group than in the control group.

**Conclusion:**

Compared with conservative treatment, surgical treatment can prolong hospital stay to some extent. However, it has the advantages of more rapid healing and lessened pain. For rib fractures in elderly individuals, surgical treatment is a safe and effective option under strict surgical indications and is recommended.

## 1. Introduction

Rib fractures are the most common injuries in chest trauma. At present, there is no complete Chinese database of chest trauma patients, and it is difficult to obtain accurate data on chest trauma. It is conservatively estimated that the number of patients with rib fracture is 1.5 million to 2 million per year (1). With population ageing, the proportion of middle-aged and elderly rib fracture patients is increasing yearly (2).

Compared with younger patients, elderly rib fracture patients have a higher incidence of complications and mortality (3, 4). Furthermore, the morbidity and mortality of elderly patients with flail chest are significantly higher than in younger patients and they have higher nursing costs (5, 6). Elderly rib fracture patients, especially those with flail chest, are at high risk for complications such as pneumonia, prolonged ventilator use, prolonged hospital stay, and chronic debilitating pain after discharge (7). Previous studies have shown that flail chest and severe pulmonary complications are important mortality risk factors in elderly rib fracture patients (3, 8, 9).

At present, the treatment of rib fracture in elderly patients generally consists of either conservative treatment or surgical treatment (10). Conservative treatment mainly includes symptomatic treatment, such as analgesia, assisted respiratory therapy, anti-infection therapy and chest plate fixation. However, the prognosis can be poor because elderly patients often have more underlying diseases, low body resistance and a high incidence of complications (3, 11). The surgical stabilization of rib fractures (SSRF) is a surgical procedure in which a fractured rib is immobilized to reduce pain and promote recovery of body function (12, 13). Previous studies have shown that SSRF improves outcomes, such as reducing the mortality, complication rate, and duration of mechanical ventilation and intensive care unit (ICU) stay (14, 15). However, there is a lack of systematic research on the treatment of rib fracture in elderly individuals, and the level of evidence in existing studies is relatively low. All confounding factors such as age and various fracture complications need to be considered and excluded to determine whether surgical treatment can improve patient outcomes (16, 17). The objective of this study was to evaluate whether SSRF can positively affect the outcome of rib fractures in elderly patients by comparing surgical vs. conservative treatment in terms of hospital admission, mortality, posttreatment symptom relief and rib fracture healing.

## 2. Materials and methods

### 2.1. Setting

The Beijing Trauma Burn Rescue Center is part of Beijing Jishuitan Hospital and receives trauma patients from the Beijing region and throughout the country. The technical level of thoracic surgery is at the forefront in the country, with an annual outpatient volume of more than 10,000 and an annual emergency volume of more than 1,500. The hospital has rich experience in the treatment of trauma patients with thoracic trauma combined with other conditions.

### 2.2. Patients

A total of 881 elderly rib fracture patients admitted to the thoracic surgery department of Beijing Jishuitan Hospital from 2013 to 2020 were initially screened for inclusion. A total of 703 elderly patients were selected and divided into the surgery group (268 cases) and the control group (435 cases) according to the surgical stabilization of rib fractures. The inclusion criteria were as follows: (1) age ≥60 years and (2) diagnosis of acute rib fracture based on medical history and imaging evidence. The exclusion criteria were as follows: (1) patients lost to follow-up (64 cases, 7.26%); (2) patients with severe injuries other than chest, that is, patients with abbreviated injury scale (AIS) score ≥3 for the head and neck, face, abdomen, limbs or body surface (114 cases, 12.94%).

Due to significant differences in the basic characteristics of the two groups after study inclusion (Table 1), they could not be compared. Therefore, the propensity score matching method was used to match the two groups of patients. After matching, 121 patients were included in both the surgery and control groups, and there were no significant differences in the basic characteristics of the two groups (Table 1).

**Table 1:** Comparison of baseline characteristics between the surgery and control group before and after matching.

Baseline characteristic	Before matching			After matching		
	Surgery group (*n* = 268)	Control group (*n* = 435)	*p* value	Surgery group (*n* = 121)	Control group (*n* = 121)	*p* value
Age, years	66.46 ± 5.51	68.03 ± 5.86	0.000	66.95 ± 5.70	67.17 ± 5.64	0.760
Male/female	156/112	213/222	0.017	65/56	62/59	0.699
**Underlying diseases**						
COPD	3	10	0.260	1	1	1.000
Asthma	1	5	0.277	1	1	1.000
Diabetes	29	73	0.029	13	15	0.688
Cerebrovascular diseases	81	157	0.110	41	36	0.490
Smoking	90	135	0.482	40	37	0.679
Number of fractures	6 (4–8)^a^	5 (3–7)	0	6 (4–8)	6 (4–7)	0.687
Flail chest	122	74	0.000	49	38	0.141
**Fracture complications**						
Pleural effusion	199	307	0.291	88	85	0.669
Pneumothorax	85	122	0.300	35	35	1.000
Atelectasis	80	123	0.655	41	34	0.331
**AIS score**						
Head and neck	0 (0–0)	0 (0–0)	0.195	0 (0–0)	0 (0–0)	0.572
Face	0 (0–0)	0 (0–0)	0.859	0 (0–0)	0 (0–0)	1.000
Chest	4 (3–5)	3 (3–4)	0.000	3 (3–5)	3 (3–4)	0.874
Abdomen	0 (0–0)	0 (0–0)	0.974	0 (0–0)	0 (0–0)	0.873
Limbs	0 (0–2)	0 (0–2)	0.026	0 (0–2)	0 (0–2)	0.525
Body surface	1 (0–1)	0 (0–1)	0.001	0 (0–1)	1 (0–1)	0.202
ISS	16 (9–24.75)	13 (9–16)	0.000	14 (9–21)	14 (9.5–20)	0.427
Admission VAS score	9 (8–9)	7 (6–8)	0.000	8 (7–9)	8 (7–9)	0.393

COPD, chronic obstructive pulmonary disease; AIS, abbreviated injury scale; ISS, injury severity score; VAS, visual analogue scale.

^a^ Quantitative data with a nonnormal distribution were analysed as medians and quartiles [M (p_25_ - p_75_)].

### 2.3. Treatment methods

**Figure 1:**
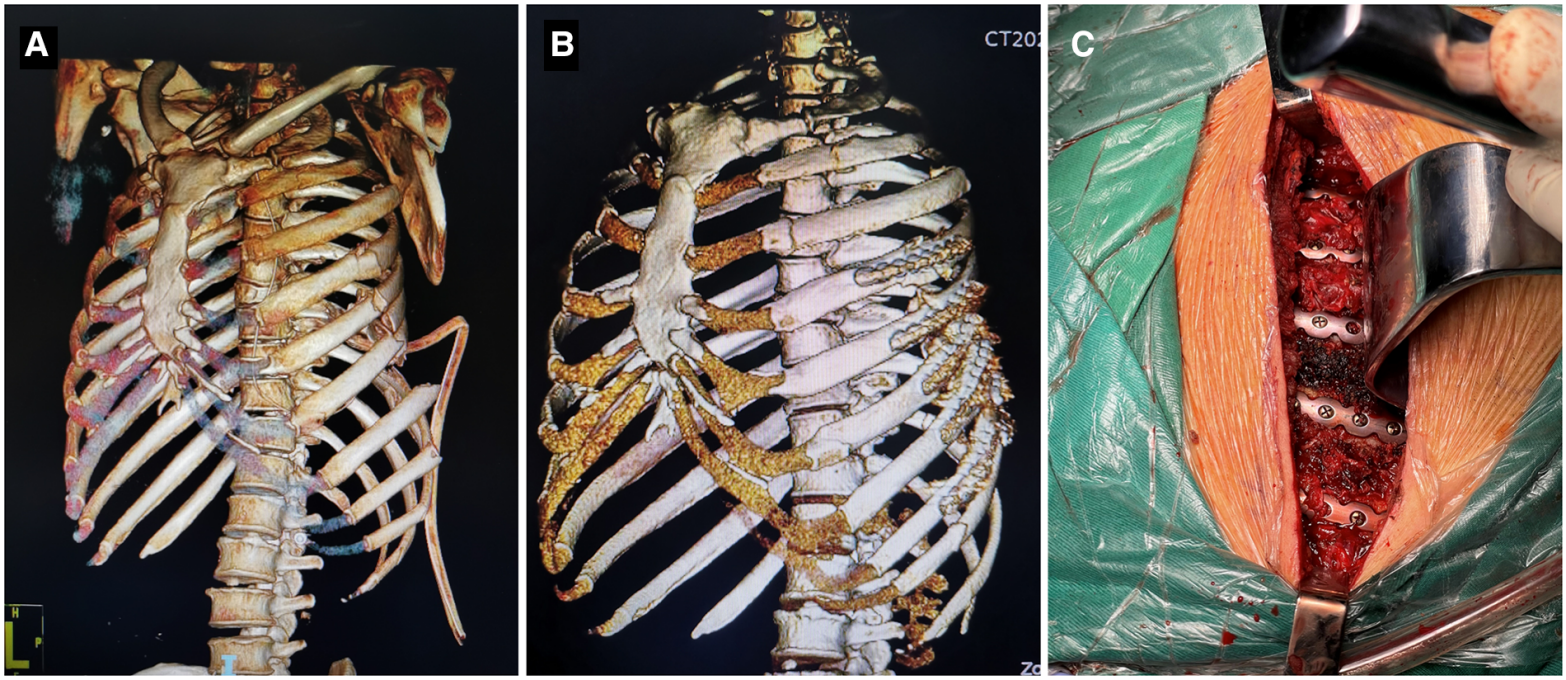
Internal fixation of a multiple rib fracture. A 57-year-old male with multiple traumas: traumatic hemothorax; flail chest with fractures of the left ribs 1–10 and right rib 5; bilateral pulmonary infection; bilateral pulmonary contusions. (**A**) CT 3D reconstruction prior to surgery suggested multiple fractures ribs. (**B**) Postoperative CT 3D reconstruction suggested that the multiple fractures ribs were repositioned and the internal fixation was well positioned. (**C**) Intraoperative photo showing after placement of internal fixation.

Surgical treatment: The surgical treatment was surgical stabilization of rib fractures (SSRF), performed under general anesthesia, with all patients in supine or decubitus position. preoperative ultrasonography was used to locate the broken end of the rib fracture and select the appropriate incision approaches according to the rib fracture site and the anatomical characteristics of the chest wall. The incision length is usually between 5 cm and 15 cm. The surgical steps were as follows: cutting the skin, separating the muscle layer along the muscle space, exposing the broken ends. The fracture was fixed by placing a new pure titanium U-shaped plate (MatrixRIB, Johnson & Johnson, Shanghai, China) at the broken end and ensured that there were 2–3 fixation nails on both sides of the broken end (Figure 1). The wound was rinsed with normal saline and hemostatic materials and devices were used to fully stop the bleeding. A drainage tube was placed in and the incision was sutured layer by layer and bandaged with pressure. postoperative analgesia pump was used routinely to relieve the pain of fractured ribs. On the first day after surgery, 10 mg of penzosin was injected, followed by long-term oral administration of Imericoxib tablets 0.1 g, BID.

Conservative treatment: Conservative treatment included the usage of rib straps or chest plates to immobilize the thorax, oxygen supplementation, sputum atomization, pain reliever, and management of fracture complications. Patients with pulmonary infection were given anti-inflammatory therapy. Closed thoracic drainage was performed for patients with massive hemothorax and/or pneumothorax. Patients with severe lung injury were treated with mechanical ventilation. Long-term oral administration of Imericoxib tablets 0.1GM BID for analgesia.

### 2.4. Observation indicators

(1) The admission status of the patient: length of hospital stay, length of ICU stay, and duration of mechanical ventilation; (2) The number of hospital deaths; (3) Delayed symptoms after treatment: fracture healing rate (follow-up for 9 months), fracture healing time, reduction of pain score, and duration of pain medication use. The evaluation of pain relief and the fracture healing index included the pain score and follow-up fracture healing. All patients were evaluated for the pain score on admission and at discharge. The usage and duration of pain medications were followed up after discharge.

**Figure 2:**
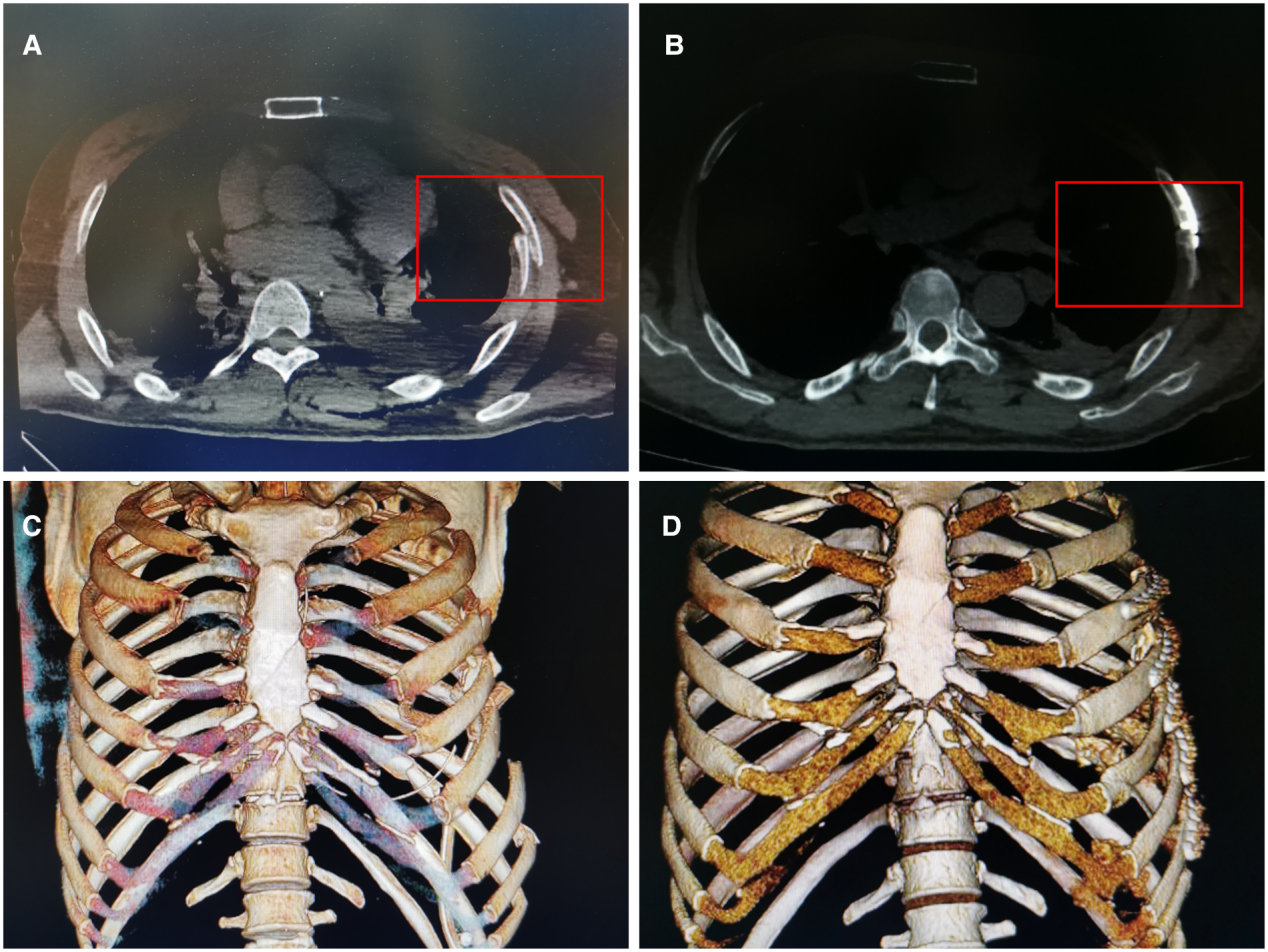
CT scan images of a patient with rib fracture. The same patient as Figure 1. (**A**) Preoperative CT scan suggested a left rib fracture. Inside the red box is the broken ends. (**B**) Three months after surgery, CT axial plain scan at the same level showed that the left rib fracture healed well, there was continuous callus formation at the fracture in the red box. (**C**) Preoperative CT 3D reconstruction suggested multiple fractures ribs. (**D**) Postoperative CT 3D reconstruction suggested good repositioning of the broken ends and the internal fixation.

Fracture healing was followed up after discharge, mainly assessed by imaging examination, physical examination and clinical symptoms. In this study, we selected a CT scan to examine the healing of the rib fracture because we could clearly see the fracture line on the axis of CT (Figure 2). In both the surgery group and the control group, we ask them to review the CT every 4 to 6 weeks until CT showed that the fracture had healed or the fracture had not healed 9 months after the injury. Nine months after injury, a patient with a nonunion rib fracture on CT was recorded as nonunion.

### 2.5. Research methods

This was a cohort study. Baseline information of the surgery and control groups included patient age, sex, underlying diseases (chronic obstructive pulmonary disease, asthma, diabetes, cardiovascular and cerebrovascular disease), smoking, number of fractures, flail chest, fracture complications (pleural effusion, pneumothorax, and atelectasis) judged by imaging changes, AIS score, injury severity score (ISS) score and pain score upon admission. With these basic characteristics as predictive variables, the calliper value was set as 0.005, and the ratio was 1:1. We calculated the bias matching score of each case using the logistic regression equation, and cases with similar scores in the two groups were matched. After matching, we compared the observation indices between the 242 patients in the surgery and control groups to analyse differences in the efficacies of these two treatment methods for acute rib fracture in elderly individuals.

### 2.6. Statistical analysis

SPSS version 26 (SPSS Inc., Chicago, IL, USA) was used for data analysis. Quantitative data with a normal distribution are expressed as the mean ± standard deviation (*X* ± SD), and an independent sample t test was used for comparisons between groups. Quantitative data with a nonnormal distribution were analysed as medians and quartiles [*M* (*p*_25_–*p*_75_)], and the Mann‒Whitney *U*-test was used for comparisons between groups. The Wilcoxon signed-rank test was used for comparison of paired quantitative data. Qualitative data were expressed as the frequency, and the *χ*^2^ test was used for comparisons between groups. *p* < 0.05 was considered to be statistically significant.

## 3. Results

Table 1 shows the baseline characteristics of the included patients before treatment. Before matching, there were no statistically significant differences in underlying diseases, smoking, fracture complications, or AIS scores of the head, neck, face and abdomen between the 268 patients in the surgery group and the 435 patients in the control group before matching (*p* > 0.050). However, they were significantly different in terms of age, sex, diabetes, number of fractures, flail chest, AIS scores for the chest, limbs and body surface, ISS, and admission pain score (*p* < 0.050). After matching, none of the baseline conditions were significantly different between the two groups (*p* > 0.050).

Of the 242 patients after matching, 98.8% had multiple rib fractures (239 cases), 36.0% had flail chest (87 cases), 52.9% had minor injuries (128 cases), 35.9% had serious injuries (87 cases), and 11.2% had severe injuries (27 cases). According to the visual analogue scale (VAS) score, 88.4% (214) of the patients had severe pain.

To reduce the impact of underlying diseases and injury severity as confounding factors on the evaluation of therapeutic efficacy, we performed a 1:1 matching of age, sex, underlying diseases, smoking, fracture severity and complications in the two groups of patients. After matching, there were no significant differences in these basic conditions between the two treatment groups or within a group before treatment (Table 1). The matching calliper value of the propensity score in this study was very small, indicating a high degree of fit between the patients. Therefore, the results of comparing the efficacy of the two treatment methods is considered to be reliable.

### 3.1. Comparison of treatment

There was no significant difference in the length of ICU stay or mechanical ventilation duration between the surgery group and the control group (*p* > 0.050); however, the length of hospital stay was significantly longer in the surgery group than in the control group (*p* < 0.050) (Table 2). Table 3 shows the length of hospitalization of the different groups with different baseline conditions after matching. In general, the length of hospitalization stay in the two groups with different conditions was significantly different (*p* < 0.050), except that women, those ≥75 years old, and those without pleural effusion did not show significant differences (*p* > 0.050).

**Table 2:** Comparison of hospitalization parameters between the surgery group and the control group.

Clinical indicator	Surgery group (*n* = 121)	Control group (*n* = 121)	*p* value
Length of hospital stay (*X* ± SD)	11.39 ± 4.21	9.48 ± 4.08	0.000
Days in ICU (*X* ± SD)	5.38 ± 3.05	6.38 ± 5.41	0.222
Mechanical ventilation duration (*X* ± SD)	77.20 ± 55.32	104.09 ± 100.13	0.231

*X*, mean; SD, standard deviation; ICU, intensive care unit.

**Table 3:** Comparison of length of hospital stay among bassline characteristic groups.

Group	Number of patients		Length of hospital stay (X ± SD)		*p* value
	Surgery group	Control group	Surgery group	Control group	
All	121	121	11.39 ± 4.21	9.48 ± 4.08	0.000
Male	65	62	12.09 ± 4.28	9.74 ± 4.09	0.002
Female	56	59	10.57 ± 4.02	9.20 ± 4.07	0.073
<75	109	110	11.41 ± 4.38	9.36 ± 3.62	0.000
≥75	12	11	11.17 ± 2.29	10.64 ± 7.41	0.815
Yes	49	38	13.04 ± 4.54	10.58 ± 4.42	0.013
No	72	83	8.98 ± 3.82	10.26 ± 3.58	0.033
Yes	88	85	12.30 ± 4.36	10.19 ± 4.45	0.002
No	33	36	8.97 ± 2.57	7.81 ± 2.29	0.051
Yes	35	35	12.29 ± 4.73	9.74 ± 3.64	0.014
No	86	86	11.02 ± 3.95	9.37 ± 4.25	0.009
Yes	41	34	12.49 ± 3.69	9.65 ± 4.13	0.002
No	80	87	10.83 ± 4.37	9.41 ± 4.07	0.032

### 3.2. In-hospital mortality

The in-hospital mortality was 0.83% in the surgery group and 3.30% in the control group. There was no significant difference in the in-hospital mortality between the two groups (*p* > 0.050) (Table 4).

**Table 4:** Comparison of hospital mortality between the surgery group and the control group.

	No. deaths	No. survivals	Overall	Mortality (%)	*p* value
Surgery group	1	120	121	0.83	0.175
Control group	4	117	121	3.30	
Overall	5	237	242	2.1	

### 3.3. Symptom relief after treatment

After 9 months of follow-up, the fracture healing rate was 96.67% in the surgery group and 88.89% in the control group. There was a significant difference in the healing rate (Tables 5, 6) and healing time (Table 7) after treatment in all patients and in flail chest patients (*p* < 0.050). The fracture healing rate in the surgery group was significantly higher than in the control group (*p* < 0.050). Additionally, the healing time in the surgery group was significantly shorter than in the control group (*p* < 0.050).

**Table 5:** Comparison of fracture healing between the surgery group and the control group 9 months after treatment.

	Incomplete healing	Complete healing	Overall	Healing rate (%)	*p* value
Surgery group	4	116	120	96.67	0.020
Control group	13	104	117	88.89	
Overall	17	220	237	92.83	

Five of the 242 patients died—so the total number was 237.

**Table 6:** Comparison of fracture healing 9 months after treatment between the surgery group and the control group among flail chest patients.

	Incomplete healing	Complete healing	Overall	Healing rate (%)	*p* value
Surgery group	2	46	48	95.83	0.022
Control group	7	28	35	80.00	
Overall	9	74	83	89.16	

**Table 7:** Comparison of fracture healing time- improvement in pain score- and duration of pain medication use between the surgery and control groups.

Clinical indicator	Surgery group (*n* = 121)	Control group (*n* = 121)	*p* value
Healing time [*M*(*p*_25_–*p*_75_)] − *m*	3 (3–3)	3 (3–7)	0.000
Improvement of pain score [*M*(*p*_25_–*p*_75_)] − *m*	4.5 (3.0–5.0)	2.0 (2.0–3.0)	0.000
Duration of pain medication use [*M*(*p*_25_–*p*_75_)] − *m*	1 (1–2)	2 (1–3.75)	0.000

We performed the Wilcoxon signed–rank test to analyse the pain scores of the 242 patients. There was a significant difference between inpatient and outpatient visits (*p* = 0.000), and the pain score was lower after surgical treatment. There were significant differences in the improvement of pain score and the duration of pain medication use between the surgery group and the control group (*p* < 0.050). Pain relief was significantly better in the surgery group than in the control group, and the duration of pain medication use was significantly shorter in the surgery group (Table 7).

## 4. Discussion

According to the Chinese consensus for the surgical treatment of traumatic rib fractures 2021 (C-STTRF 2021) (1), SSRF is recommended for multiple rib fractures with flail chest (IIA). Patients without surgical contraindications and without flail chest can also benefit from surgical treatment (IIB). The consensus encourages the surgical treatment of rib fractures. Although there is no clear evidence that surgical treatment improves healing in patients, it has been proven to improve lung function (18). Additionally, an RCT study showed that it relieved pain (19), and it reduces the disability rate and time to return to work (20).

However, as a special population, there is still a lack of specific research on the evaluation of surgical treatment of rib fractures in patients over 60 years old. Rib fracture is the most common chest trauma and is often associated with adverse outcomes such as lung infection, ARDS, respiratory failure, and death (21, 22). These poor outcomes are mainly due to several reasons. First, elderly individuals often have a poor ability to repair the physical damage of rib fracture, and they often have osteoporosis. The osteogenic ability of osteoblasts is reduced in older adults; in contrast, the bone resorption capacity of osteoclasts is enhanced (23). Therefore, the quality of fracture healing is poor, and the proportion of delayed healing and bone nonunion is high (24). Second, respiratory restriction due to pain makes older individuals more at risk of adverse outcomes (25). Third, elderly patients often have poor cardiopulmonary function and underlying diseases, which are also associated with poor outcomes (26). Finally, many elderly people have taken anticoagulant drugs for a long time, which complicates surgery and makes it difficult to perform (27, 28).

Based on the diagnosis and treatment experience of our center and previous literature reports, there are significant differences in the characteristics of trauma between the elderly and young people. The main cause of injury in the elderly is blunt trauma (29). Elderly people are more likely to develop hemothorax after blunt trauma (1). The elderly is frailer, leading to higher rates of anticoagulant drug use (30). And the elderly has more complications, such as the sepsis and multiple organ failure (31). Therefore, the elderly has a higher hospital mortality rate (31). In contrast, the cause of trauma in young people is often accidents (32). Due to the relatively severe trauma, young people often have multiple injuries, and the injuries are more severe and acute (33). Such patients often experience renal injury. Therefore, special attention needs to be paid to the renal function of young patients (34). In summary, there are differences in the etiology and clinical manifestations of injuries between young and elderly people, and targeted diagnosis and treatment should be based on specific age.

This study showed that compared with conservative treatment, surgical treatment can extend the length of hospital stay to some extent, but it has advantages in terms of the fracture healing rate, fracture healing time and fracture pain relief.

Slobogean GP et al. (35) conducted a meta-analysis of previous studies and found that for conservative treatment, the lengths of hospital stay were 13, 15, 18, and 21 days; the lengths of ICU stay were 15, 18, 20, and 21 days; and the durations of mechanical ventilation were 13, 15–18, and 20–22 days. Surgical treatment reduced the average length of hospital stay and the average length of ICU stay after mechanical ventilation by 4.0 days, 4.8 days, and 7.5 days, respectively. There was a statistically significant difference between surgical treatment and conservative treatment, and the findings suggested that SSRF could better promote symptom relief and functional recovery in elderly patients with rib fracture compared with conservative treatment. In the present study, the lengths of hospitalization, ICU stay and mechanical ventilation in the two groups were generally shorter than the above values, which may be related to the quality of the treatment measures taken in our centre or the exclusion of patients with severe head and abdominal complications.

We found that there was no significant difference in the length of ICU stay or mechanical ventilation between the two groups. However, the length of hospitalization was significantly longer in the surgery group than in the control group. We analysed the baseline factors that may have affected the length of hospital stay. Except for the pleural effusion group, the length of stay in the surgery group was significantly longer than in the control group regardless of whether the patients had flail chest, pneumothorax or atelectasis before surgery. This may be related to the poor recovery ability of elderly individuals after surgery. A recent meta-analysis by Choi J (36) showed that compared with younger patients, elderly patients have a higher incidence of osteoporosis and significantly higher postoperative complications of rib fracture, and they are more prone to plate fracture, displacement and infection after surgery.

Few datasets on rib fractures in elderly individuals have been available for previous studies. Nevertheless, previous studies have found that the overall mortality for patients with rib fracture ranges from 0.0% to 33.3%, compared with 0.0% to 30% for patients who received SSRF and 0.0% to 57.9% for those who received nonsurgical treatment (10, 37, 38). These findings indicate that surgical treatment can significantly reduce patient mortality (3). A meta-analysis by Cataneo AJ (8) found that there was no significant difference in mortality between surgery and conservative treatment groups in patients with rib fractures. The causes of death were often pneumonia, pulmonary embolism, mediastinum, and septic shock. However, Shibahashi K et al. (18) pointed out that the sample size of this meta-analysis was small (*n* = 123) and therefore the correlation analysis was not reliable. In our study, the mortality rate decreased significantly after sample matching, indicating that the mortality results are indeed affected by the sample size. Mortality in both groups was very low, especially in the surgery group, which had a lower mortality than the control group, but the difference was not statistically significant.

Marasco S et al. (19) found that the fracture healing rates (including complete union and partial union) of both fixed and unfixed bone ends were very high in flail chest patients 3 months after SSRF treatment (88.46% and 86.54%, respectively), but there was no significant difference between the two groups. In the present study, the fracture healing rates of the surgery group and the control group were also rather high 9 months after surgery, at 96.67% and 88.89%, respectively. The fracture healing rates of the flail chest group and the control group were also as high as 95.83% and 80.00%, respectively. However, we found that the fracture healing rate of the surgery group was significantly higher than that of the control group. The fracture healing time of the surgery group was 3 months on average, while that of the control group was 3–7 months. The healing time of the surgery group was significantly shorter than that of the control group. The ribs move with breathing, and if not fixed, the broken ends of the fracture will move in response to breathing or coughing owing to shear and axial movements (39). Compared with axial motion, shear motion has a higher torsion stress. Shear motion delays the healing of the diaphysis, while axial motion promotes healing. In addition, shear motion affects the angiogenesis process in the periosteum healing tissue (40).

Patients in the surgery group were significantly better than those in the control group in terms of the improvement in pain score and the duration of pain medication use (41). This study showed that SSRF more significantly and rapidly relieved pain symptoms in the elderly patients compared with conservative treatment. However, surgical treatment did extend the hospital stay. Therefore, the surgical treatment of SSRF has pros and cons—it can accelerate patient recovery and improve their quality of life, but it may extend the length of hospital stay and increase the risk of hospitalization during the perioperative period. Doctors should carefully evaluate a patient's condition and surgical indications and the risks and benefits of surgery to choose the optimum treatment (38, 42, 43).

As for the cost of treatment, given the advantages of the surgical group, pain improvement with fewer drugs, less ICU and mechanical ventilation need to make this treatment option more cost-effective. But on the other hand, the cost of surgical implants, surgical procedures, anesthesia, occupational therapy services and initial specialized equipment, as well as consumables, may be a major barrier for acceptance of this technique.

Based on a large amount of previous clinical experience, our centre has developed indications for the selection of surgery to treat elderly patients with rib fracture. At present, our centre recommends SSRF for elderly patients with flail chest and who have no contraindications to surgery. For non-flail chest patients with two or more rib fractures and obvious displacement of the broken ends in more than half of the fractures, surgery is recommended. For patients with non-flail chest with complications, conservative analgesia and respiratory management should be routinely used. If not effective, patients with early severe pain and well-aligned broken fracture ends without obvious surgical contraindications are recommended for surgical treatment. Patients with severe complications should be treated with positive pressure ventilation by an endotracheal intubation ventilator. For patients with poor conservative treatment, respiratory deterioration and long-term chest floating, if there is no obvious contraindication, physicians should consider SSRF.

There are some limitations to this study. The number of patients was greatly reduced after matching, which may have influenced the results. This study was a single-centre study, and the results showed that the length of hospital stay, length of ICU stay, mechanical ventilation duration, and mortality were all lower than in previous studies. As a primary trauma and burn treatment centre located in Beijing, we receive critical chest trauma patients from all over the country, and our institute is a leader in surgical quality, ICU quality and nursing quality in China. Therefore, the results may not be generalisable to other treatment centres. To more objectively evaluate the curative effect of SSRF in China, studies are needed with larger sample sizes and that include more centres.

With the development of surgical techniques and the improvement of quality of life, age is no longer an absolute contraindication for surgical treatment. We hope that surgical treatment can provide better options for elderly patients and improve patients' quality of life. Of course, surgery itself is traumatic, especially for the elderly, so the length of hospital stay after surgery will be prolonged, but the mortality rate of patients does not increase, and the long-term recovery of patients also shows a better effect, we hope that through our efforts can provide more excellent treatment methods for the treatment of patients with rib fracture.

## 5. Conclusion

In conclusion, we studied the efficacy of two treatment methods for elderly rib fracture patients using the propensity score matching method. We found that surgical stabilization of rib fractures extended the length of hospital stay compared with conservative treatment, but it had advantages in terms of the fracture healing rate, healing time, and pain relief. For rib fractures in elderly individuals, surgical treatment does not significantly increase mortality when applied under strict surgical indications.

## Data Availability

The data analyzed in this study is subject to the following licenses/restrictions: The datasets generated and/or analysed during the study are available from the corresponding author on reasonable request. Requests to access these datasets should be directed to zhangqiang3046@126.com.
